# A comparative evaluation of measures to assess randomness in human-generated sequences

**DOI:** 10.3758/s13428-024-02456-7

**Published:** 2024-07-01

**Authors:** Tim Angelike, Jochen Musch

**Affiliations:** Institute of Experimental Psychology, Department of Psychological Assessment and Differential Psychology, Heinrich Heine University Düsseldorf, Universitätsstraße 1, 40225 Düsseldorf, Germany

**Keywords:** Randomness, Human random number generation, Algorithmic complexity, Entropy

## Abstract

Whether and how well people can behave randomly is of interest in many areas of psychological research. The ability to generate randomness is often investigated using random number generation (RNG) tasks, in which participants are asked to generate a sequence of numbers that is as random as possible. However, there is no consensus on how best to quantify the randomness of responses in human-generated sequences. Traditionally, psychologists have used measures of randomness that directly assess specific features of human behavior in RNG tasks, such as the tendency to avoid repetition or to systematically generate numbers that have not been generated in the recent choice history, a behavior known as cycling. Other disciplines have proposed measures of randomness that are based on a more rigorous mathematical foundation and are less restricted to specific features of randomness, such as algorithmic complexity. More recently, variants of these measures have been proposed to assess systematic patterns in short sequences. We report the first large-scale integrative study to compare measures of specific aspects of randomness with entropy-derived measures based on information theory and measures based on algorithmic complexity. We compare the ability of the different measures to discriminate between human-generated sequences and truly random sequences based on atmospheric noise, and provide a systematic analysis of how the usefulness of randomness measures is affected by sequence length. We conclude with recommendations that can guide the selection of appropriate measures of randomness in psychological research.

**Supplementary Information:**

The online version contains supplementary material available at 10.3758/s13428-024-02456-7.

Psychologists have long been interested in the human ability to generate random-like sequences (Baddeley, 1966; Falk & Konold, 1997; Wagenaar, 1972). The basic consensus is that humans generally do not behave randomly but instead exhibit systematic patterns that make their decisions predictable (Bocharov et al., 2020; Schulz et al., 2012; Shteingart & Loewenstein, 2016). Previous studies have used various measures from psychological research, computer science, and mathematics to quantify randomness or the lack thereof (Gauvrit et al., 2016; Ginsburg & Karpiuk, 1994; Oomens et al., 2015, 2021; Towse & Neil, 1998). However, the heterogeneity in the plethora of measures used across studies has been criticized by researchers for being subjective and not allowing for comparisons between studies (Barbasz et al., 2008; Gauvrit et al., 2014, 2016), with the earliest criticisms dating back decades (Wagenaar, 1972). In this paper, we provide the first large-scale comparison of a diverse collection of randomness measures in terms of their ability to discriminate between random and human-generated sequences. We undertook this investigation to provide practitioners with data-based recommendations for selecting appropriate measures of randomness in psychological research.

The ability to generate random-like sequences is typically assessed using so-called random number generation (RNG) tasks, in which participants are asked to generate a sequence of random numbers. Typically, participants are asked to use numbers in the interval from 1 to 9 (Capone et al., 2014; Jokar & Mikaili, 2012; Miyake et al., 2000; Schulz et al., 2021; Zabelina et al., 2012), but there are also experimental paradigms that require the use of 10 or more numbers (Ginsburg & Karpiuk, 1994; Peters et al., 2007; Towse, 1998; Towse & Cheshire, 2007) as well as studies with only two numbers (Biesaga et al., 2021; Biesaga & Nowak, 2024; Gauvrit et al., 2016). Another variant of this task requires participants to produce a random sequence of letters (Baddeley, 1966; Cooper et al., 2012; Larigauderie et al., 2020). Typically, participants are carefully instructed about the properties of random sequences to avoid measuring participants’ misconceptions about randomness (Schulz et al., 2021; Towse & Cheshire, 2007).

The study of human RNG is of interest in several areas of psychology because it can be used to better understand various cognitive functions. For example, RNG performance can be used as an indicator of working memory and inhibitory capacity (Friedman & Miyake, 2004; Heuer et al., 2005) or, more generally, of the central executive (Cooper, 2016; Miyake et al., 2000; Miyake, Friedman et al., 2001a). Cognitive models have been developed to explain how humans attempt to generate random-like sequences of numbers (Cooper, 2016). Because RNG tasks are demanding and draw on different cognitive resources, they are often used as secondary load tasks to analyze performance on both primary and secondary tasks in dual-task experiments. Studies have shown a significant reduction in performance on the primary task and in the randomness of the sequences generated in the secondary load task (Howarth et al., 2016; Knott & Dewhurst, 2007; Miyake, Witzki et al., 2001b). RNG tasks have also been used to study the cognitive abilities of patients with psychiatric and neurological disorders such as schizophrenia (Peters et al., 2007; Shinba et al., 2000) or acquired brain injury (Maes et al., 2011), who show even more stereotyped behavior than healthy controls as evidenced by the tendency to generate series of adjacent number pairs (e.g., 7-6 or 4-5). The ability to generate random-like sequences has been found to develop similarly to other cognitive abilities across the lifespan: it increases from childhood to adolescence, peaking at age 25, followed by a decline that becomes steeper around age 60 (Gauvrit et al., 2017).

To study how different cognitive processes contribute to the ability to generate random-like sequences, researchers need measures that are sensitive to systematic patterns that people may exhibit. The problem that needs to be addressed is that, in principle, it is impossible to tell with certainty from a given sequence whether it was generated by a random or a deterministic process. An apparently systematic sequence such as 1-2-1-2-1-2-1-2 has the same probability of occurrence under a random process as the less systematic sequence 1-1-2-2-2-1-1-2 (Gauvrit et al., 2016). However, we can infer from the occurrence of systematic patterns in the first example sequence that it is more likely to have been generated by a deterministic, nonrandom process than the second sequence. Approaches to assessing the randomness of sequences generated in an RNG task are based on the identification of systematic patterns that provide evidence that a nonrandom process could have generated them (Gauvrit et al., 2016; Ginsburg & Karpiuk, 1994; Towse & Neil, 1998). Researchers have not agreed on a single way to assess randomness, as evidenced by the heterogeneity among approaches summarized in the following section. Instead, the measures used to quantify randomness or the lack thereof can be broadly grouped into three categories.

## Approaches to the detection of deviations from randomness

### Measures commonly used in psychological research

Over the past several decades of research, psychologists have used a variety of measures to assess randomness. Many of these measures take into account biases in human behavior, such as the fear of repetition, which refers to the tendency to avoid direct repetitions of a number (Cooper, 2016). Two commonly used collections of randomness measures for analyzing human-generated random number sequences were proposed by Ginsburg and Karpiuk (1994) and Towse and Neil (1998). Ginsburg and Karpiuk proposed a collection of 10 measures that assess typical human biases in an RNG task: the coupon score, the gap score, the poker score, the runs index, the cluster ratio, the RNG index, diagram repetitions, repetitions, series, and variance of digits. These measures were often aggregated using principal component analyses based on the correlation between randomness measures across participants. These analyses revealed three components: cycling, seriation, and repetition. The cycling component is shown by the tendency to select numbers that have not been used recently with increased probability; the seriation component is shown by the tendency to stereotype behavior, such as the tendency to ascend or descend a series of numbers; and the repetition component reflects the avoidance of direct repetition (Peters et al., 2007).

Towse and Neil’s work is a refinement of Ginsburg and Karpiuk’s work, removing redundant measures, such as the poker score, the cluster ratio, diagram repetition, repetitions, and series, and adding new ones, such as the phi score—a measure of repetition that, unlike most of the measures discussed in Ginsburg and Karpiuk (1994), can be computed over any interval length—and the redundancy index, the turning point index, and the adjacency score. Towse and Neil also performed a principal component analysis on all of their measures. They concluded that the correlation between measures of randomness across participants was best explained by a four-component solution. Towse and Neil (1998) named these four components “equality of response usage,” “short repetitions,” “prepotent associates,” and “long repetitions.” Their “equality of response usage” component mirrors Ginsburg and Karpiuk’s cycling component in that both components reflect behavior that is aimed at using all numbers in a sequence in an equal manner. This is also evidenced by other randomness measures that load on these components, such as the coupon and the median gap score, which assess how long it takes for all numbers to occur once and how long it takes, on average, for a number to be repeated. The component “prepotent associates” assesses whether there is a tendency to engage in stereotypical behavior, such as repeating some pairs of number more often than others in a sequence, which is conceptually similar to the seriation component. Randomness measures that assess the tendency to repeat number pairs load on both of these components in the Ginsburg and Karpiuk and Towse and Neil studies. The original repetition component of Ginsburg and Karpiuk (1994) was found in two components reflecting the number of repetitions over short and long distances. The generation of a number that has been used one to three numbers before in the sequence is an example of a short-distance repetition; the generation of a number that has been used at least four numbers before is an example of a long-distance repetition. The Towse and Neil collection comes with the RgCalc software tool and has recently been implemented in the R package randseqR (Oomens et al., 2021). The measures proposed by Towse and Neil are widely used in psychological research (Barbasz et al., 2008; Cooper, 2016; Larigauderie et al., 2020; Linschoten & Harvey Jr., 2004; Maes et al., 2011; Schulter et al., 2010; Zabelina et al., 2012). Some of the measures included in Towse and Neil’s collection that assess whether some responses or pairs of responses are generated more frequently than others in a sequence, such as the redundancy index, the RNG index, and the RNG2 index, fall into a second category of entropy-based measures that can also be used to quantify the randomness of a sequence.

### Block entropy

As defined by Shannon (1948), entropy is a measure that quantifies the amount of information in a sequence based on the frequency distribution of the symbols that make up the sequence. A sequence in which each symbol is equally frequent has maximum entropy and contains the most information. A sequence consisting of only one symbol has entropy of 0 and contains no information due to redundancy. When applied to the study of human RNG tasks, entropy indicates whether there is an uneven distribution of the frequency of numbers in a sequence. In this context, low entropy would indicate that a person has used some numbers more than others in a sequence. Block entropy is an extension of standard Shannon entropy that goes beyond the analysis of individual numbers by assessing whether there is inequality in the frequency of blocks (also called *n*-grams) of consecutive responses in a sequence (Moore et al., 2018; Shannon, 1948). For example, a sequence might contain the block 7-4-1 more frequently than all other blocks of the same size, resulting in lower block entropy. Some of the measures in Towse and Neil’s (1998) collection that are used to assess whether there is inequality in responses or pairs of responses in a sequence are variants of Shannon and block entropy. However, to our knowledge, measures of block entropy that assess whether there is inequality in response triplets or quadruples (or even larger blocks) have not been systematically used to analyze human RNG tasks. Including such measures based on information theory may provide a more complete assessment of recurring patterns in human RNG that are associated with the seriation and “prepotent associates” components in Ginsburg and Karpiuk (1994) and Towse and Neil (1998), respectively. Recent findings indicate that individuals may differ strongly in their tendency to repeat specific number blocks larger than two numbers (Schulz et al., 2021). However, measures of randomness that are traditionally employed in psychological research do not account for blocks of numbers larger than two.

### Measures of algorithmic complexity

Recently, some promising new measures of randomness have been proposed that originate from algorithmic complexity theory (Gauvrit et al., 2014; Zenil et al., 2018). These randomness measures are based on the Kolmogorov–Chaitin definition of complexity (Gauvrit et al., 2016). In this framework, complexity is defined as the length of the shortest computer program that can produce a given object—in this case, a sequence of numbers. A sequence that follows a systematic pattern, such as 1-2-1-2-1-2-1-2, can be generated by a program that follows a simple algorithmic rule (e.g., repeat response pair 1-2 four times). This sequence would therefore be considered not complex and probably not generated by a random process. However, the same sequence would be considered random according to classical Shannon entropy if, when choosing from two possible responses, both responses (e.g., 1 and 2) occur equally often. The concept of algorithmic complexity is important for algorithms for lossless compression of long sequences, where the goal is to replace the original sequence with a shorter sequence that contains all of the information of the original sequence but replaces recurring patterns (Lempel & Ziv, 1976). A sequence is considered more random the less compressible it is (i.e., the fewer repeating patterns it contains). Measures of Lempel–Ziv complexity have only rarely been used in psychological research. However, the study by Wong et al. (2021) is an example of such an application. When analyzing human-generated sequences, psychologists might profit from also considering measures of algorithmic complexity. A recent investigation found a strong correlation between the algorithmic complexity of a sequence and its randomness as perceived by humans (Gauvrit et al., 2016). Therefore, measures of algorithmic complexity may help to assess specific regularities that humans often perceive as random (Falk & Konold, 1997; Gauvrit et al., 2016) and that may therefore be present in sequences humans produce in RNG tasks.

The software needed to approximate the algorithmic complexity for short sequences has only recently been implemented (Gauvrit et al., 2014, 2016; Soler-Toscano et al., 2014). It is based on the coding theorem method, according to which sequences with low algorithmic complexity have a high probability of being produced by a deterministic process (in this case, a computer program), and sequences with high algorithmic complexity have a low probability of being produced by a deterministic process. The computer programs used to quantify the probability that a deterministic process produces a sequence are Turing machines. A Turing machine is a theoretical model of a general-purpose computer that produces an output based on a specified set of rules. By sampling from many Turing machines, it is possible to construct a frequency table showing how often a sequence is generated by a randomly drawn Turing machine and, thus, by a deterministic process. This information is then used to approximate the algorithmic complexity of a sequence by taking the negative logarithm of the relative frequency with which the sequence is generated. Higher algorithmic complexity indicates sequences that are rarely generated by a deterministic process, and lower algorithmic complexity indicates less complex sequences that are frequently generated by a deterministic process. This approach promises to detect systematic patterns for short sequences (≤ 12), which no previous measure of randomness has been able to do. It was extended to longer sequences by Zenil et al. (2018), who proposed the block decomposition method (BDM) by combining it with the concept of entropy, which allows the repeated use of identical blocks of numbers in a sequence to be penalized. The usefulness of measures of algorithmic complexity has already been demonstrated for the analysis of binary sequences in a psychological setting (Biesaga et al., 2021; Biesaga & Nowak, 2024; Gauvrit et al., 2016), but not for sequences of more than two possible numbers.

## The present study

We present the first large-scale comparison of a diverse collection of randomness measures from psychology (Ginsburg & Karpiuk, 1994; Oomens et al., 2021; Towse & Neil, 1998) and from information theory and algorithmic complexity theory (Gauvrit et al., 2016; Lempel & Ziv, 1976; Shannon, 1948; Zenil et al., 2018). We determine how well randomness measures are suited to determining whether a human or a random process generated a sequence. Thus, our approach focuses on identifying measures of randomness that best detect biases that humans exhibit when they attempt to generate random numbers. Which measures best detect evidence of systematic behavior that is not present in random sequences? The rationale behind this validation approach is that a measure that is sensitive to nonrandomness, i.e., systematic patterns exhibited by humans, should be able to discriminate between random and human-generated sequences with high confidence.

As a gold standard for comparison, we used data from atmospheric noise as a presumably truly random source (Furutsu & Ishida, 1961; Haahr, 2023). We chose atmospheric noise to avoid having to rely on computer-generated pseudorandom sequences based on deterministic algorithms as a validation criterion. Unlike pseudorandom number generators, true random number generators such as atmospheric noise produce random numbers that are aperiodic and nondeterministic (Haahr, 2023).

In addition, we investigate how the length of a sequence affects the usefulness of measures to discriminate between human-generated and random sequences, as sequence lengths often vary widely across studies (Figurska et al., 2008; Ginsburg & Karpiuk, 1994; Schulz et al., 2021). Moreover, it is plausible to assume that the measures differ in their sensitivity to detect systematic patterns in human-generated sequences depending on the sequence length, as some measures, such as the complexity measure of Gauvrit et al. (2016), were specifically designed for short sequences, while other measures, such as compression algorithms, are generally only used to analyze longer sequences (Zenil et al., 2018). Based on our results, we try to derive practical recommendations for the selection of the most useful measures for the analysis of randomness in human behavior.

## Methods

### Design

Sequences of numbers were generated either by human participants in an RNG task or by continuously updated variations in the amplitude of atmospheric noise data provided by the website random.org (Haahr, 2023) as accessed through an interface included in the R package *random* (Eddelbuettel, 2017). For each measure of randomness, we computed the resampled correct classification rate at which sequences of numbers could be correctly assigned to their generating source (human vs. random). We used logistic regression models with each measure of randomness as the independent variable and the source of the sequences as the dependent variable. Each logistic regression model was bootstrapped (*n* = 1000). To this end, each of the *n* logistic regression models was computed on 1660 randomly sampled sequences (with replacement). The sample size for each bootstrap iteration was chosen to equal our human sample size (*n* = 830) multiplied by 2, since there were as many random as human-generated sequences. Drawing samples with replacement allowed for the possibility of repeated occurrences or no occurrences at all of a sequence in the training set used for computing the logistic regression model. Each model’s predictive performance could then be evaluated on the sequences that were not part of the training process, resulting in *n* correct classification rates for each measure of randomness that could then be used for constructing 95% confidence intervals. Going beyond previous approaches, we also present the first comprehensive investigation of how sequence length affects classification rates. To this end, we calculated the correct classification rate for complete sequences and for the first 20, 50, and 100 digits of the 200-digit sequences.

### Material

#### Random number generation task

##### Instructions

Following the approach of Schulz et al. (2021) and Towse and Cheshire (2007), we instructed participants to consider the following essential features of random number generation: (1) equal probability of responses, (2) independence of responses from each other, and (3) absence of patterns and unpredictability of responses. We explained the RNG task using the analogy of repeatedly drawing a number from 1 to 9 from a hat, returning the drawn number, and then shuffling the contents of the hat to repeat this procedure. We provided participants with examples of repetitive patterns to avoid and an example of what a random sequence might look like. We asked participants to generate a random number each time they heard a metronome tone. If they missed a response, they were instructed to move on and generate another number with the next sound.

##### Experimental task

We used a 3 × 3 grid to record the responses, with a number from 1 to 9 displayed in each cell of the grid. The numbers 1, 2, and 3 were in the first row, 4, 5, and 6 were in the middle row, and 7, 8, and 9 were in the bottom row (in the order of their naming). The experimental paradigm of using a 3 × 3 grid for the RNG task was adopted from Maes et al. (2011), who showed that the use of this grid yielded similar results with respect to the widely used Towse and Neil (1998) collection of randomness measures as when the numbers were produced orally. Before the start of RNG task, participants had to confirm through the click of a button that they were ready to do the task. Following this confirmation, the screen cleared for 2000 ms, after which a horizontally centered 3 × 3 grid of 450 × 450 pixels appeared. After a further 1000 ms, the rhythmic sound of a metronome began and was repeated every 1500 ms until the RNG task was completed. Participants were instructed to randomly select and click on one of the cells with their mouse each time they heard the metronome sound. In response, the selected cell changed its color to orange for 250 ms, providing visual feedback to the participant. For the 1000 ms following their selection, participants could not select another cell from the grid, ensuring that they could not speed through the experiment. Once the last trial of the task was completed, the grid disappeared, and the study moved to the next page. Participants had to complete 200 trials of the RNG task, which took exactly 5 minutes if they kept to the rhythm of the metronome. A green bar indicated their overall progress on the RNG task.

### Procedure

The study was conducted using the online platform Unipark (https://www.unipark.com/). Participants were welcomed on the first page of the study. We informed them about the general purpose of the study (random number generation), their rights, and the intended use of their data in order to obtain their informed consent. On the following page, we asked for demographic information about their age, gender, German language proficiency, and educational level. Next, participants had to complete an audio check to ensure that they could hear the metronome during the RNG task. They had to listen to a short audio file in which they heard a rooster, and then choose which of several animals they had heard. On the next page, participants were given instructions on how to complete the RNG task. Instructions could only be skipped after 60 seconds, so clicking through the instructions was not possible. We then asked participants two simple multiple-choice questions to make sure they understood the instructions. The first question asked how they should behave during the experiment (answer: randomly). The second question asked when participants should choose a random number (answer: at the sound of the metronome). Participants were excluded from the study at this stage if they answered one or both questions incorrectly. On the next two pages, participants were allowed to adjust the volume of the metronome so that the sound was comfortable, and then completed 10 test trials of the RNG task to become familiar with it. This phase was followed by the main experimental task, which consisted of 200 trials. After completing the experimental task, participants provided self-reports on a need for cognition scale (Lins de Holanda Coelho et al., 2020), a conscientiousness scale (Rammstedt et al., 2013), and on their mathematical abilities (e.g., school grade in mathematics, learning stochastics in school or at university). Additionally, participants could enter comments about this study. On the next page, they were asked to indicate whether they were serious about participating in the study (Aust et al., 2013), with assurances that this question would not result in forfeiting their compensation. On the last page, participants were debriefed and thanked for their participation. Participants could again enter comments in a text box for feedback, as they now knew the purpose of the study. The median time it took to complete the study was 12 minutes.

### Sample

Participants were recruited through the online panel Bilendi (https://www.bilendi.de). Participants had to be at least 18 years old and be native German speakers or have a comparable language level to participate in the experiment. Participants could take part in the study using a desktop computer, a tablet (with a touchscreen), or a laptop. Another requirement of the study was that participants were able to play an audio stream on the device they were using to participate. Participants who did not correctly answer both comprehension questions about the experimental paradigm on the first attempt (*n* = 181) were not allowed to continue at this stage due to presumed inattention, in order to ensure the quality of the data. The total sample consisted of 830 participants, as 21 participants had to be excluded for the following reasons: one participant reported using a 10-sided fair dice for the task; another participant used the same answer 50 times in a row (for a quarter of the task); 16 participants indicated in a seriousness check at the end of the study that they did not participate sincerely; one participant used only three of the nine possible numbers during the entire RNG task; two participants did not follow the rhythm of the metronome in the RNG task (median intertrial latency over 2000 ms). The final sample consisted of 405 men, 424 women, and one person who reported a non-binary gender. The age of the sample ranged from 18 to 87 years (*M* = 51.15, *SD* = 15.37). Most participants, 567, reported a certificate of secondary education or a high school diploma as their highest level of education, 245 participants had a college degree, 16 had obtained a Ph.D., and only two participants had not completed high school. The participants were compensated with €0.50 for their participation in the study. To increase the motivation of the participants, we conducted a lottery and awarded an additional bonus of €5 to the 30 participants who generated the most random sequences according to the coupon score (the participants did not know how we would determine the most random sequences). This was to provide an additional incentive for participants to be as random as possible in the RNG task. The lottery was announced at the end of the instructions for the RNG task.

### Measures of randomness

#### Commonly used measures in psychological research (Towse & Neil, 1998)

We computed the most widely used collection of randomness measures in psychological research, namely that of Towse and Neil (1998), who also described these measures in detail in their review. To compute these measures, we used the R package randseqR as described in Oomens et al. (2021). For the computation, we used the *randseqR option* (same name as the package) as suggested by the authors of the package. Thus, for measures that rely on computing the frequency of occurrence of response pairs in a given sequence, the last response pair consisting of the last and (after starting over) the first sequence number was not considered.

##### Redundancy index

The redundancy index is a measure of whether there is inequality in the frequency of responses in a sequence, approaching 100 if a sequence consists of only one response and 0 for perfect equality of all possible responses. The redundancy index is a transformed version of the classical Shannon entropy.

##### Random number generation (RNG) index

The RNG index measures whether pairs of responses in a sequence (e.g., 4-1, 1-5, and 5-6 in the sequence 4-1-5-6) are equally distributed, given the underlying frequency distribution of the first response in a pair. Thus, the RNG index is a measure of whether the transition probabilities from one response to another are equal. The index ranges from 0 (perfect equality of transition probabilities) to 100 (all transition probabilities are either 1 or 0).

##### RNG2 index

The RNG2 index follows the same logic as the RNG index. However, instead of looking at the transition probabilities between consecutive responses, it computes whether there is inequality in transition probabilities between interleaved responses by a gap of 1. For example, in the sequence 4-1-5-6, the two pairs 4-5 and 1-6 are considered. The range of this index is identical to the regular RNG index.

##### Null-score quotient (NSQ)

The NSQ is the proportion of response pairs that do not occur in a sequence relative to the number of possible response pairs. The measure is multiplied by 100 to obtain percentages. The range of this measure is from 0 to 100, with a value of 0 indicating that all possible response pairs occur in a sequence. For this measure, lower values indicate a more even distribution of response pairs and therefore a higher degree of randomness.

##### Coupon score

The coupon score measures how long it takes for all possible responses in a sequence to occur. This measure is computed by iterating over a sequence and counting the time until each response has occurred at least once. The result is stored, and the procedure starts again with the first response after the completed set. The final score is the average of the lengths required to observe all of the responses. If a sequence does not contain all possible responses, the score is set to the length of the sequence + 1. Whether a particular score indicates low or high randomness can be judged only by comparing it to the average score of random sequences, because the average time it takes for all responses to appear depends strongly on the cardinality of the set of available numbers. For this comparison, we used the sequences based on atmospheric noise data, which we used as a benchmark for a random source of numbers.

##### Repetition gap

The repetition gap is the average gap between identical responses in a sequence. We computed three variants of this measure: the mean, median, and mode over the distribution of gaps between identical responses. Like the coupon score, the repetition gap can be interpreted as a measure of randomness only when compared to the mean repetition gap of random sequences.

##### Adjacency index

The adjacency index measures the proportion of ascending pairs relative to the total number of pairs in a sequence. The measure can be computed for ascending (e.g., 3-4) and descending pairs (e.g., 7-6), or a combination of both. The index is multiplied by 100 to represent percentages. On average, a random sequence will contain a percentage of adjacent pairs equal to the proportion computed by dividing the number of possible adjacent pairs by the number of all possible response pairs.

##### Turning point index

Turning points are defined as minima and maxima in a sequence (e.g., the sequence 1-3-5-4-3-7 has two turning points, 5 and 3). The number of observed turning points is then compared to the theoretically expected number of turning points and multiplied by 100. Values of random sequences for this measure range from 90 to 100 (Oomens et al., 2021). Higher values indicate more turning points than theoretically expected, and lower values indicate fewer turning points than theoretically expected.

##### Runs index

The runs index computes the variance over the lengths of ascending subsequences in a sequence. For instance, the sequence 1-4-7-3-5 contains two runs, one of length 3 (1-4-7) and one of length 2 (3-5). The runs index is the variance computed over the two values 3 and 2, which represent the run lengths. The idea behind this measure is to capture the variability in the length of ascending subsequences. A higher value would indicate frequent switching between short and long runs of ascending numbers, and a value of 0 would indicate that all runs of ascending numbers in a sequence have the same length. This measure must also be compared to the expected value of randomly drawn sequences in order to interpret an observed value as random or not.

##### Phi index

The phi index is a measure of repetitions of responses that are divided by a gap of other responses between them. More specifically, the measure counts the number of repetitions between the first and the last response of all blocks of specified length in a sequence and compares this frequency to the expected frequency of repetitions based on the observed number of repetitions between the first and last response of blocks that are one response smaller. Negative values indicate too few repetitions, and positive values indicate more repetitions than theoretically expected. We computed the phi index for blocks ranging in size from 2 to 10 to allow comparability with other measures computed over different block sizes.

### Block entropy

Block entropy is a measure that indicates whether there is inequality in blocks of responses in a sequence. Blocks are determined by iterating over the sequence with a rolling window of size *k*. We defined *k* to be between 2 to 10, excluding only blocks of size 1, as the redundancy index in the previous section is a transformed version of Shannon’s entropy (Shannon, 1948), which reduces to block entropy of size 1. High values of block entropy indicate an equal distribution of blocks of length *k*; low values indicate inequality, with a minimum of 0 indicating that a sequence consists of only one response. We chose a block size of 10 as a cutoff to allow comparison with complexity measures for short sequences, which only allow block sizes of up to 10 to be considered (see below).

### Measures of algorithmic complexity

For clarity, we divided the group of algorithmic complexity measures into three subgroups: averaged algorithmic complexity measures for short sequences as proposed by Gauvrit et al. (2016), the block decomposition method as proposed by Zenil et al. (2018), and compression algorithms (Lempel & Ziv, 1976).

#### Averaged algorithmic complexity for short sequences

This measure was computed using the R- package *acss* by Gauvrit et al. (2016), which is based on the coding theorem method. Complexity was computed over a rolling window for each block of length *k* between 2 and 10. A block size of 10 was chosen as the cutoff, because for block sizes 11 and 12, for nine possible values in a sequence, the complexity could not be computed for all possible sequences (Gauvrit et al., 2016). Finally, the mean of all complexity values was taken as an aggregate measure for the entire sequence.

#### Block decomposition method (BDM)

The BDM (Zenil et al., 2018) was also computed over a rolling window for each block size *k* between 2 and 10. Each block was then assigned its algorithmic complexity from the previous section. However, instead of repeatedly counting the algorithmic complexity of recurring blocks, the total score is only increased by the logarithm of the frequency of a block after its first occurrence. A repetition of blocks is thus penalized by the BDM formula.

#### Compression algorithms

We also computed two different compression algorithms: Lempel–Ziv complexity (Lempel & Ziv, 1976; LZ76) following the guidelines of Kaspar and Schuster (1987) and Dolan et al. (2018), and the gzip algorithm using the *memCompress*() function in R programming language (R Core Team, 2023). The goal of compression algorithms is to search for repeating patterns in a sequence and replace them with a symbol representing that pattern. In this way, the length of a sequence can be reduced without losing information, since the original sequence can be reconstructed from the new compressed version of the sequence. This approach can also be used to test how random a sequence is, since random sequences without patterns should be difficult to compress, while systematic sequences with many repeating patterns should result in shorter compressed sequences.

## Results

Data analysis was performed using the R environment for statistical computing version 4.3.0 (R Core Team, 2023). The following additional packages were used for the analysis: acss 0.3-2 (Gauvrit et al., 2016), randseqR 0.1.0 (Oomens et al., 2021), randfindR 0.1.0 (Angelike, 2022), papaja 0.1.1 (Aust & Barth, 2022), ggplot2 3.4.2 (Wickham, 2016), and ggpubr 0.6.0 (Kassambara, 2020). The data and code used in all analyses can be found at https://osf.io/xwzup/.

### Computation of randomness indices

First, we drew random sequences equal in length (200 digits) and number (830 participants) to the experimental data. For this purpose, we used the R package *random* (Eddelbuettel, 2017)*,* which is an interface to the random.org website that generates data sequences of numbers based on atmospheric noise data (Haahr, 2023). Next, we computed all of the randomness measures summarized in the methods section over all sequences (human-generated and random). We also computed all measures over the first 20, 50, and 100 numbers of each sequence to examine the effect of sequence length on these measures. To ensure the robustness of our findings, we repeated all analyses with two additional random datasets^1^. The first additional dataset served as an independent replication and was also drawn from the R package *random* that provides access to atmospheric noise data. The second additional dataset was generated using the pseudorandom number generator in R programming language with the sample() function. The results did not vary across datasets, confirming their robustness, with all findings remaining virtually the same. For the sake of shortness and clarity, only the results based on the first random dataset generated from atmospheric noise are reported here.

Neither gender nor any of the personality variables showed a significant association with the measures of randomness under investigation, after a Bonferroni correction was applied to avoid inflating the alpha error. Participants who had studied stochastics at school or had received higher education tended to have higher randomness scores, even after a Bonferroni correction, but their level of randomness was still significantly lower than that of truly random data generated by atmospheric noise.

### Classification results

In this section, we investigated which measures of randomness were best suited for discriminating between human-generated and random sequences. For this purpose, we constructed logistic regression models for each individual measure of randomness where the score of a measure computed over all sequences was the independent variable, and the binary dependent variable was the source of generation of a sequence (human or random). The analysis was repeated for each randomness measure computed over all sequence lengths examined. To control for possible effects of overfitting, the correct classification rate of each model was determined through bootstrapping (*n* = 1000; further details are provided in the design section). This allowed us to construct empirical confidence intervals of the correct classification rate for each randomness measure by selecting the 2.5th and the 97.5th percentiles of the bootstrapped correct classification rates. If the confidence intervals of two different randomness measures do not overlap, the difference between these two measures regarding the correct classification rate can be considered significant. For example, suppose a hypothetical randomness measure A has an upper bound of 75% for the correct classification rate at the 97.5th percentile, while randomness measure B has a lower bound of 80% at the 2.5th percentile. Since in this case the confidence intervals for the correct classification rates of these two measures do not overlap, we can conclude that randomness measure B is better suited for distinguishing between human-generated and random sequences than measure A. Similarly, we considered a measure as showing only approximate random performance if a confidence interval included the value .50.

### Overview of measures

**Fig. 1:**
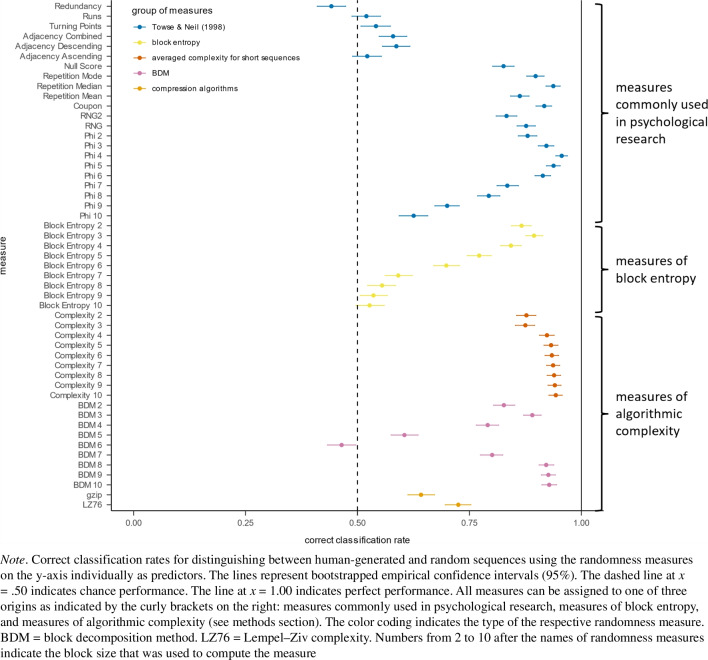
Correct classification rate for randomness measures ordered by their group of origin

First, we provide an overview of the usefulness of the measures computed over the entire sequences in discriminating between human-generated and random sequences (see Fig. 1 for visualization and the Supplementary Materials for all raw values). The correct classification rate was high for many randomness measures (*M* = 0.78, with a range from 0.44 to 0.96). Thus, on the basis of many randomness measures, it was possible to distinguish between human-generated and random sequences. Overall, measures of averaged algorithmic complexity were consistently useful for discriminating between the two sources of sequences (between .88 and .94), although the correct classification rate increased with block size. Similarly, the phi index showed a high correct classification rate (.63 to .96, highest for a block size of 4). Interestingly, the results showed a higher range for the BDM and block entropy (.46 to .93 and .53 to .89, respectively). BDM measures showed high correct classification rates for larger block sizes of 7 to 10 (.80 to .93) and for smaller block sizes of 2 to 4 (.79 to 89) but not for moderate block sizes of 5 to 6 (.60 and .46). Block entropy measures were most useful for block sizes of 2 to 4, with decreasing performance as block size length increased. Other useful measures for distinguishing between human-generated and random sequences were the coupon score (.92) and all variants of the repetition gap score, with the median gap between identical numbers being the most useful (.94). The LZ76 showed significantly better than chance performance (.73) but fell short of other measures of algorithmic complexity. The runs, redundancy, and turning point index, as well as all variants of the adjacency index, showed, at best, slightly above-chance performance in distinguishing between human-generated and random sequences (.44 to .59).

### Groups of randomness measures

A complete collection of all randomness measures, including their descriptive values and correct classification rates by sequence length, can be found in the Supplementary Materials. This analysis is divided into five sections: measures commonly used in psychological research (Towse & Neil, 1998) using the R package randseqR (Oomens et al., 2021), block entropy measures, and complexity measures, with the last being divided into measures of algorithmic complexity for short sequences, the BDM, and compression algorithms.

#### Common measures in psychological research

**Fig. 2:**
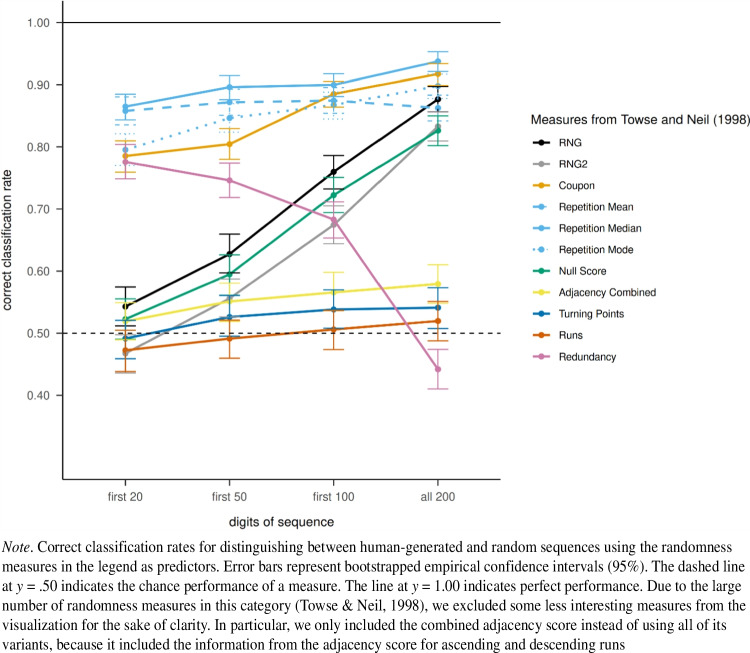
Correct classification rate into human-generated and random sequences of logistic regression models based on the measures of Towse and Neil (1998)

The median repetition gap and the coupon score showed a high correct classification rate with only small effects of sequence length on performance (see Fig. 2). The mean and mode over the repetition gap between identical pairs were also among the most useful measures for distinguishing between human-generated and random sequences, although they performed slightly worse. The RNG and RNG2 indices and the NSQ showed only chance or near-chance performance for short sequences (size 20), but became increasingly useful for distinguishing between human-generated and random sequences as the sequence length increased. These measures assess systematic repetition in response pairs, and apparently require longer sequences to show clear differences between human-generated and random sequences. The combined adjacency, runs, and turning point indices did not show high correct classification rates regardless of sequence length. One striking finding was that the redundancy index enabled adequate discrimination between human-generated and random sequences for the first 20 digits (.78), but this performance declined for longer sequences (.44 for the complete sequences). The redundancy index is a measure that assesses whether all possible responses (here, the numbers from 1 to 9) are equally likely to occur. In this experiment, human-generated sequences showed greater response equality than random sequences during the first 20 numbers of the sequence (see Supplementary Materials). This effect disappeared in the long run, until there was no difference between the groups in the relative frequency of the numbers. Humans may show too much equality in the frequency of their responses, which is particularly evident for short sequences (Ginsburg, 1997). This observation is also consistent with previous findings that people may try too hard to use all responses equally compared to random sequences of the same length (Ginsburg & Karpiuk, 1994).

**Fig. 3:**
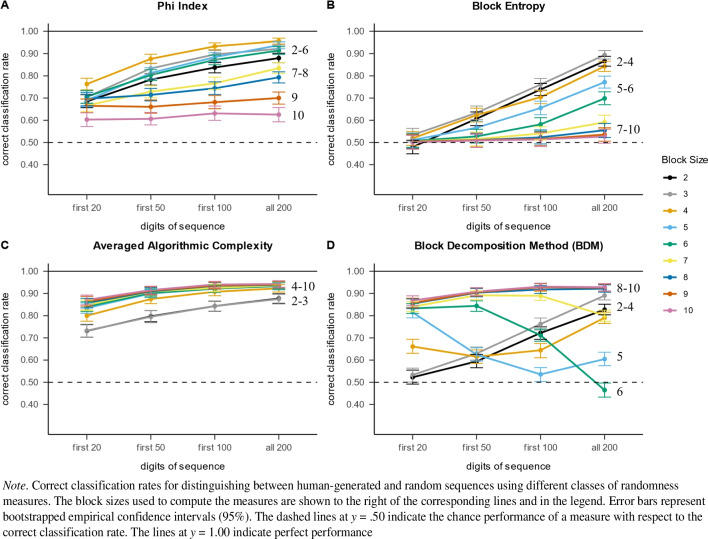
Correct classification rate into human-generated and random sequences of logistic regression models based on measures computed over block sizes 2 to 10

The correct classification rate obtained with the phi index increased steadily the longer the sequence used for computing the measure. Performance was particularly high when computing the measure for blocks of size 4, although the correct classification rate was also high for block sizes 2 to 6 (see Fig. 3A). The correct classification rate was not as high for larger block sizes.

#### Block entropy

When examining block entropy, two interesting results can be highlighted (see Fig. 3B). First, all measures of block entropy were uninformative for analyzing short sequences, as indicated by the near-chance performance in distinguishing between human-generated and random sequences using only the first 20 numbers of a sequence. Second, increasing the length of the sequence used to compute block entropy increased the correct classification rate into human-generated and random sequences. However, the magnitude of this increase seemed to depend on the block size. A clear increase in the correct classification rate can be seen for block sizes of 2 to 4. Block sizes of 5 to 6 yielded moderate increases in the correct classification rate, while block sizes of 7 to 10 were only marginally informative in terms of distinguishing between human-generated and random sequences for any given length of a sequence. This was probably due to the exponentially increasing number of distinct blocks with increasing size (${9}^{k},$ where *k* is the block size). Measures of block entropy look for inequality in the use of blocks of a given size, which is particularly hard to find when the number of possible blocks is too large. Block entropy measures for large block sizes (e.g., 9 or 10) are likely to require much longer sequences to be informative.

#### Measures of algorithmic complexity

The section on measures of algorithmic complexity is divided into three sections: measures of algorithmic complexity for short sequences, the BDM, and compression algorithms.

#### Averaged algorithmic complexity for short sequences

In this section, we investigate the measures of algorithmic complexity as proposed by Gauvrit et al. (2016). Figure 3C shows that the correct classification rate for all measures of averaged algorithmic complexity increases steadily with the length of the sequence considered and the block size used to compute the measure. Overall, measures of averaged algorithmic complexity showed a consistently high correct classification rate compared to all other measures, with the lowest correct classification rate above 70% and the highest above 90%.

Note that we observed a rather surprising result for all measures of averaged algorithmic complexity. Higher values are associated with more complex and random sequences. However, we found that human-generated sequences had higher values for these measures than sequences generated by a random process (see Supplementary Materials). This finding seems to be caused by the short block length of numbers for which it can be computed. In this study, the measure was computed for blocks up to size 10. A high-complexity sequence usually contains all possible numbers equally often, as can be seen in the examples of high-complexity binary strings in Gauvrit et al. (2014). However, if there are nine possible numbers, high-complexity sequences will appear as if someone had cycled through all available numbers when generating the sequence, because each number will occur approximately once. As a result, short sequences of high complexity may resemble those generated by humans, who show such a cycling tendency in their behavior. This is illustrated by the negative correlation between algorithmic complexity for block size 10 and the coupon score, *r*(828) = −.59, *p* < .001, showing that participants with a stronger tendency to cycle through all available numbers (lower coupon score) obtain higher values of algorithmic complexity.

#### BDM

Results for BDM did not exactly follow the pattern of the averaged algorithmic complexity (see Fig. 3D). For large block sizes (8 to 10), the BDM was useful for distinguishing between human-generated and random sequences. However, for block sizes of 5 to 6, the correct classification rate decreased steadily with increasing sequence length. On the other hand, for block sizes of 2 to 4, the correct classification rate using the BDM was at chance level when computed over the first 20 numbers of the sequence but increased steadily with longer sequences.

**Fig. 4:**
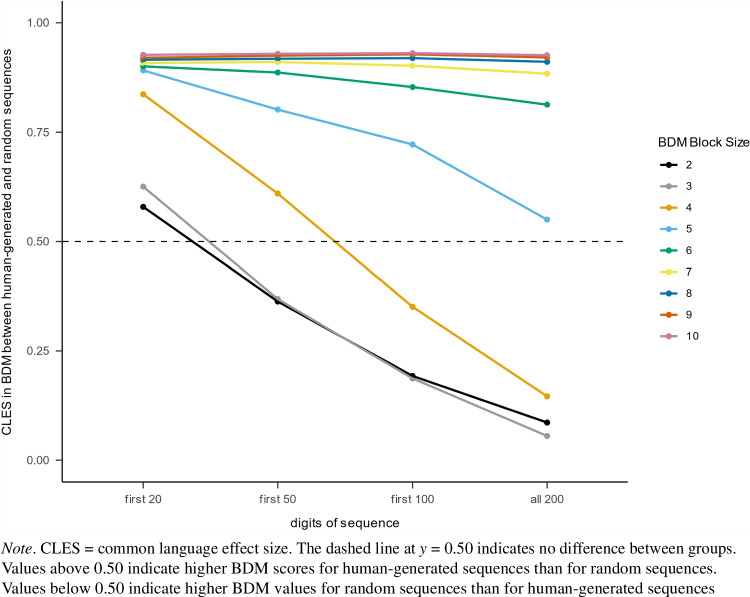
Difference in BDM scores between human-generated and random sequences measured by the common language effect size (CLES)

To further investigate this finding, we computed the common language effect size for the difference in BDM scores between human-generated and random sequences, which indicates the probability that a randomly selected BDM score from the human sample is higher than a randomly selected BDM score from the random sequence sample (Fig. 4). The results showed a general tendency: as sequence length increased, the differences between BDM scores for human-generated and random sequences decreased. For block sizes of 2 to 4, this difference even reversed, so that random sequences had higher BDM scores than human-generated sequences. For larger block sizes, however, humans had consistently higher BDM scores regardless of sequence length. The reason for this pattern of results probably lies in the combination of algorithmic complexity and entropy in the BDM. Humans generally show a tendency to cycle through all available numbers too quickly, leading to higher averaged algorithmic complexity on the one hand and a tendency to repeat smaller blocks of size 2 to 4 on the other hand. Penalizing the latter leads to lower BDM scores for human-generated sequences for smaller block sizes. However, penalizing repetitive patterns becomes increasingly ineffective with increasing block size due to the exponentially increasing number of distinct blocks, as explained in the section on block entropy. As a result, human-generated sequences have higher BDM scores than random sequences for larger block sizes and lower scores for smaller block sizes. For moderate block sizes (5 to 7), the combination of these opposing effects may explain the declining performance of the measure as these effects appear to cancel each other out, resulting in smaller differences in BDM scores between human-generated and random sequences.

#### Compression algorithms

Both compression algorithms, LZ76 and gzip, showed a comparatively low rate of correct classification into human-generated and random sequences (see Supplementary Materials). The correct classification rate peaked at about 60–70%. This is significantly lower than the highest correct classification rate of randomness measures from each of the measure groups analyzed so far. There were several examples of measures (e.g., the RNG index, phi index, measures of block entropy, or algorithmic complexity) that exceeded the 80% or even 90% correct classification rate. This finding is not very surprising given that compression algorithms are typically used to quickly compress longer sequences, such as files, and not to analyze human-generated sequences of a few hundred digits or less (Gauvrit et al., 2016; Zenil et al., 2018).

## Discussion

The present study is the first large-scale integrative comparison of a broad collection of different measures of randomness. We analyzed not only measures that are traditionally used in psychological research (Towse & Neil, 1998), but also classical measures from information theory, such as block entropy (Moore et al., 2018; Shannon, 1948), as well as measures of algorithmic complexity (Gauvrit et al., 2016; Lempel & Ziv, 1976; Zenil et al., 2018). In addition, we analyzed how the effectiveness of measures for identifying human behavior may depend on sequence length. We also proposed a classification-based approach to evaluate randomness measures in terms of their usefulness in identifying human behavior in RNG tasks. For this analysis, we did not rely on numbers obtained through pseudorandom generation from a computer for comparison; instead, we used sequences from a random source that are aperiodic and nondeterministic (Haahr, 2023).

Our results show that several measures of randomness can distinguish between human-generated and random sequences with a high correct classification rate of > .80. This is not too surprising, given the large body of research showing that humans generally fail to behave randomly (Bocharov et al., 2020; Figurska et al., 2008; Ginsburg & Karpiuk, 1994; Montare, 1999; Schulz et al., 2021). However, some randomness measures were particularly good at distinguishing between human-generated and random sequences. Complexity measures such as averaged algorithmic complexity for larger block sizes (especially block size 10), block entropy for shorter to moderately long block sizes (especially block size 3), and the phi index for moderately long block sizes (especially block size 4) were most useful. The median repetition gap score and the coupon score also showed large differences between human-generated and random sequences. We argue that researchers who wish to use measures of randomness that are sensitive to systematic patterns typical of humans should use these measures.

It should be noted, however, that the sensitivity of randomness measures to systematic patterns, which are often generated by humans, depends on the length of the sequence over which the measures are computed. Measures such as algorithmic complexity for blocks of size 10 or the repetition median already showed large differences between human-generated and random sequences for short sequences of length 20, with a rate of correct classification between human-generated and random sequences close to 90%. If researchers want to analyze short sequences or subsequences of longer human-generated sequences, they should use these two measures. The phi score (block size 4) and the coupon score were also sensitive to differences between human-generated and random sequences for short sequences, but to a lesser extent than the averaged algorithmic complexity and the repetition median.

Averaged algorithmic complexity, the median repetition gap, the phi score, and, to a lesser extent, the coupon score also showed a high correct classification rate for longer sequences, demonstrating their applicability in various contexts of RNG tasks. On the other hand, measures such as block entropy showed almost no difference at all between human-generated and random sequences of short length. These measures required sequences 100 digits or more in length to achieve a high correct classification rate for moderate block sizes. Even for sequences of 100 digits, the correct classification rate between human-generated and random sequences by block entropy could not exceed the classification rate of the average algorithmic complexity for sequences of length 20. A similar effect was observed for the RNG and RNG2 indices, which are modified measures of block entropy for number pairs. Several studies using these measures were based on the analysis of human-generated sequences of 100 digits (Friedman & Miyake, 2004; Ginsburg & Karpiuk, 1994; Maes et al., 2011; Miyake et al., 2000, Miyake, Friedman et al., 2001a; Peters et al., 2007; Towse, 1998; Zabelina et al., 2012). Thus, it would seem advisable to increase the sequence length of the RNG task if researchers plan to use these measures, as longer sequences appear to be required to exploit their full potential. Our results suggest that block entropy-derived measures for short to medium block sizes should only be used for sequences of at least, but preferably more than, 100 digits.

Several measures were not useful for identifying systematic patterns observed in humans: the turning point and the runs index showed little or no difference between human-generated and random sequences regardless of sequence length. We advise caution in using these measures in future research, as they may introduce irrelevant variance for characterizing human behavior in RNG tasks. However, this finding should be replicated in future research to determine its stability. Otherwise, our findings are consistent with previous studies such as Ginsburg and Karpiuk (1994), who found similar differences in measures such as coupon and the median repetition gap between human-generated and random-like sequences. Our results are also consistent with their finding that humans show a more even use of all possible numbers in a sequence than would be expected on average for a random sequence of the same short length (100 numbers; Ginsburg & Karpiuk, 1994).

One measure that we recommend against using for the analysis of sequences consisting of numbers in the range of 1 to 9 is the block decomposition method (BDM) due to its inconsistent interpretation. Depending on the length of the sequence and the block size used to compute it, a larger value may indicate either a randomly generated sequence or a human-generated sequence. This is likely due to the opposing effects of complexity and block entropy on scores in the BDM: on the one hand, we found that the complexity was generally higher for human-generated than for random sequences; on the other hand, the BDM formula penalizes repetitions in a similar way to block entropy, leading to lower scores for humans, especially for blocks of short to medium length. We therefore argue that researchers should use average algorithmic complexity if they wish to use complexity measures, as it consistently shows higher values for human-generated sequences than for random sequences.

A strong argument can be made against the use of compression algorithms. The investigated measures, LZ76 and the gzip algorithm, even when computed over the complete sequences, performed worse regarding the correct classification rate than the averaged algorithmic complexity as proposed by Gauvrit et al. (2016), even when the latter was computed using only the first 20 numbers of each sequence. Therefore, we cannot recommend the use of compression algorithms as measures of randomness.

How can the randomness measures considered in this investigation be used for applied research questions? Fortunately, implementations are available for all of the measures presented in this paper. The measures commonly used in psychological research proposed by Towse and Neil (1998) can be computed using either the computer program from their original publication RgCalc or the more recent implementation in the R package randseqR by Oomens et al. (2021). The algorithmic complexity of short sequences as well as the BDM can be computed using the R package acss by Gauvrit et al. (2016), who also provide an introduction and tutorial on how to use it. The BDM can also be computed using the online algorithmic complexity calculator https://complexity-calculator.com/ (Soler-Toscano et al., 2014; Zenil et al., 2018). Implementations of block entropy and LZ76 can be found in the R package randfindR at the following link: https://github.com/TImA97/randfindR (Angelike, 2022). The code used to compute all randomness measures in this investigation can be found at https://osf.io/xwzup/.

Finally, we must discuss the surprising result concerning the averaged algorithmic complexity as a measure of randomness. We found that human-generated sequences yielded higher estimates of averaged algorithmic complexity than random sequences. In this study, participants generated sequences containing the numbers 1 through 9. A highly complex sequence must contain all possible values approximately equally often, leaving little or no room for repetition if the sequence is only 10 digits in length. Consequently, a highly complex sequence with nine alternatives is a sequence that appears to show a certain cycling tendency. We argue that algorithmic complexity, as proposed by Gauvrit et al. (2014, 2016) for sequences with nine possible alternatives, does not accurately reflect randomness, since systematic nonrandom biases lead to higher values of complexity. Rather, the measure of algorithmic complexity appears to be inversely related to randomness. This limitation of the measure in terms of its interpretation needs to be addressed in future research. However, it should be emphasized that the averaged algorithmic complexity showed high sensitivity to systematic patterns that humans exhibited when attempting to generate random sequences, regardless of the sequence length, underscoring the usefulness of this measure for characterizing human behavior.

A common criticism of the state of the scientific literature on the analysis of randomness in human-generated sequences is that too many different measures of randomness are used (e.g., Gauvrit et al., 2016; Wagenaar, 1972). This makes it difficult, if not impossible, to compare the results of different studies. The goal of this paper is to inform researchers about the properties of the randomness measures they employ in their research. We analyzed a diverse collection of randomness measures in terms of their sensitivity to systematic patterns that humans show when trying to generate random sequences of numbers: measures that are motivated by psychological research (Towse & Neil, 1998), measures of block entropy (Shannon, 1948), and measures of algorithmic complexity (Gauvrit et al., 2016; Lempel & Ziv, 1976; Zenil et al., 2018). We went beyond previous research not only in the number and variety of randomness measures evaluated, but also in the systematic analysis of the influence of the sequence length on the measures’ sensitivity to systematic human behavior in RNG tasks. We showed that some measures, such as the turning point and the runs index, show only a negligible difference between human-generated and random sequences. We argue that not all of the measures proposed by Towse & Neil (1998) may be necessary for analyzing sequences from an RNG task. We found that measures such as the phi index for moderate block sizes (a measure of repetition over a number gap), the coupon score (a measure of the cycling tendency), the repetition gap score, the block entropy of shorter to moderate block sizes for longer sequences, and especially the averaged algorithmic complexity regardless of sequence length show high sensitivity to the patterns exhibited by humans in an RNG task. We hope these results help researchers to make more informed decisions about the choice of randomness measures for the analysis of RNG tasks. For a reasonably well-specified research question, only one or a few sensitive randomness measures may be sufficient, rather than a large collection of randomness measures that may contain uninformative measures.

There are many different examples of analyzing the randomness of human-generated sequences from RNG tasks. Randomization performance can be analyzed to compare different experimental groups, such as different levels of production speed (Towse, 1998), or quasi-experimental groups such as healthy versus schizophrenic patients (Peters et al., 2007) or healthy controls versus patients with acquired brain injury (Maes et al., 2011). Performance on RNG tasks has been recognized as a possible indicator of executive function (e.g., Cooper, 2016). Deterioration in this performance can, thus, be used to infer the effect of an experimental manipulation or to uncover correlates of psychiatric and neurological disorders on cognitive functions. For such purposes, it seems prudent to use measures that have been shown to be most sensitive to systematic human behavior. We hope that this study will help researchers choose the most appropriate measure of randomness for their research question. However, researchers should not be completely discouraged from using other measures of randomness if they can better answer a theoretically meaningful question. For example, Peters et al. found that patients with schizophrenia tend to respond to pairs of adjacent numbers (such as 8-7 or 1-2). This could be explicitly investigated using the adjacency score, although it did not show high sensitivity to systematic patterns found in humans in this study.

Quantifying the randomness of number sequences has applications beyond the setting of the RNG task. For example, measures of randomness could potentially be used to assess whether participants’ responses to a task or questionnaire are provided thoughtfully^2^. It would be interesting to investigate whether and which measures of randomness are most effective in distinguishing between serious and nonserious responses. For example, in a lexical decision task in which words and nonwords are presented in random order, responses that deviate substantially from a random sequence likely indicate a violation of the instruction to categorize stimuli as being words versus nonwords, and a tendency to produce some kind of systematic pattern instead. The effectiveness of measures of randomness in detecting nonserious responding could be compared with existing approaches to detect repetitive patterns that are based on computing autocorrelations between subsequent responses. These latter methods are limited to some extent because they are only sensitive to specific regularities that have sometimes been found to characterize nonserious responses (Gottfried et al., 2022). To improve data quality in empirical investigations by identifying careless responses more reliably, established measures to detect nonserious responding could be supplemented or possibly even replaced with quantitative measures of randomness.

In the present study, we did not examine measures of recurrence quantification analysis (Oomens et al., 2023) because principal component analyses show that even though only recurrence quantification analysis preserves all time-based information, recurrence quantification analysis and the measures proposed by Towse and Neil (1998) show a similar factorial structure (Oomens et al., 2015). Future research should further investigate the effectiveness of randomness measures in assessing changes in randomness over time, as these changes may reflect changes in the underlying cognitive processes (Oomens et al., 2023). Temporal changes in algorithmic complexity may improve our understanding of RNG task performance, as this measure strongly correlates with the perceived randomness of sequences (Gauvrit et al., 2016). Changes in algorithmic complexity could potentially indicate corresponding changes in information processing. Using measures of algorithmic complexity, such temporal changes have already been established for binary sequences (Biesaga et al., 2021; Biesaga & Nowak, 2024). Comparing recurrence quantification analysis and algorithmic complexity for binary sequences or for sequences based on a set size of three or more could provide additional insights into potential changes in the cognitive demands posed by RNG tasks over time. Furthermore, measures based on information theory could improve our understanding of how humans generate random numbers, as this approach has been shown to accurately reflect and to be sensitive to individual differences in pattern preferences (Schulz et al., 2021). Employing different types of randomness measures may therefore help to better understand the components underlying RNG performance.

In summary, we have compared a large collection of randomness measures for their usefulness in distinguishing between human-generated and random sequences, thereby establishing a validation criterion for judging the usefulness of a measure for identifying human behavior. These results are directly applicable to psychological research using RNG tasks. We find that some of the commonly used randomness measures are insensitive to the differences between human-generated and random sequences and are, therefore, not informative for characterizing human behavior. We also show that the sensitivity of many randomness measures can strongly depend on the sequence length used for analysis. On the other hand, some measures, such as the algorithmic complexity or the repetition gap score, showed high sensitivity to patterns indicative of human behavior for both short and long sequences. Taken together, these results can help guide practitioners in selecting the measures of randomness that are most appropriate for their research question.

### Supplementary Information

Below is the link to the electronic supplementary material.

Supplementary file1 (DOCX 66 KB)

## Data Availability

The datasets generated during and/or analyzed during the current study are available in the OSF repository, https://osf.io/xwzup/.

## Footnotes

1 We are grateful to Henrik Singmann for suggesting these additional analyses.

2 We are grateful to the editor for making this suggestion.

## Code availability

The code used to analyze the data is available in the same OSF repository as the data, https://osf.io/xwzup/.
