# Supplementary material for: A comparative evaluation of measures to assess randomness in human-generated sequences
Source: Behav Res Methods. 2024 Jul 1;56(7):7831–48. doi: 10.3758/s13428-024-02456-7 (PMC11362514; doi:10.3758/s13428-024-02456-7)
Supplement: Supplementary file 1 — Supplementary file1 (DOCX 66 KB) [file 13428_2024_2456_MOESM1_ESM.docx]

**Supplementary Materials**

*Descriptive Statistics and Correct Classification Rates Between Human-Generated and Random*

*Sequences*

| Measure | *M*_human_ | *SD*_human_ | *M*_random_ | *SD*_random_ | Correct classification rate |
| --- | --- | --- | --- | --- | --- |
| First 20 numbers of the sequences | | | | | |
| Block Entropy 2 | 4.04 | 0.21 | 4.04 | 0.15 | .48 [.45, .51] |
| Block Entropy 3 | 4.12 | 0.16 | 4.15 | 0.05 | .53 [.50, .56] |
| Block Entropy 4 | 4.07 | 0.14 | 4.08 | 0.02 | .52 [.49, .55] |
| Block Entropy 5 | 3.99 | 0.13 | 4.00 | 0.00 | .51 [.48, .54] |
| Block Entropy 6 | 3.90 | 0.12 | 3.91 | 0.00 | .51 [.48, .54] |
| Block Entropy 7 | 3.80 | 0.11 | 3.81 | 0.00 | .50 [.47, .54] |
| Block Entropy 8 | 3.70 | 0.10 | 3.70 | 0.00 | .50 [.47, .53] |
| Block Entropy 9 | 3.58 | 0.10 | 3.58 | 0.00 | .50 [.47, .54] |
| Block Entropy 10 | 3.46 | 0.09 | 3.46 | 0.00 | .50 [.47, .53] |
| RNG | 15.11 | 11.73 | 13.11 | 8.72 | .54 [.51, .57] |
| RNG2 | 12.24 | 10.43 | 11.72 | 8.01 | .47 [.44, .50] |
| Coupon | 13.78 | 4.07 | 19.41 | 2.55 | .79 [.76, .81] |
| Repetition Mean | 7.26 | 1.22 | 4.99 | 0.93 | .86 [.84, .88] |
| Repetition Median | 7.02 | 1.61 | 4.07 | 1.22 | .86 [.84, .88] |
| Repetition Mode | 5.95 | 2.75 | 2.48 | 1.80 | .80 [.77, .82] |
| Null Score | 79.92 | 1.93 | 79.93 | 1.68 | .52 [.49, .56] |
| Adjacency Asc | 9.72 | 8.77 | 9.40 | 6.39 | .49 [.46, .52] |
| Adjacency Desc | 10.66 | 7.15 | 9.77 | 6.47 | .52 [.49, .55] |
| Adjacency Combi | 20.38 | 10.59 | 19.17 | 8.64 | .52 [.49, .55] |
| Turning Points | 93.29 | 19.83 | 93.91 | 15.20 | .49 [.46, .52] |
| Runs | 0.73 | 1.35 | 0.72 | 0.56 | .47 [.44, .50] |
| Redundancy | 4.31 | 4.09 | 10.34 | 5.11 | .78 [.75, .80] |
| Phi 2 | -3.45 | 2.64 | -1.38 | 2.89 | .68 [.66, .72] |
| Phi 3 | -4.73 | 1.45 | -2.97 | 2.48 | .70 [.68, .73] |
| Phi 4 | -4.70 | 1.60 | -1.88 | 2.53 | .76 [.74, .79] |
| Phi 5 | -4.93 | 1.89 | -2.96 | 2.36 | .69 [.66, .72] |
| Phi 6 | -4.60 | 2.23 | -2.28 | 2.11 | .71 [.68, .73] |
| Phi 7 | -4.84 | 2.32 | -3.17 | 1.97 | .67 [.64, .70] |
| Phi 8 | -4.41 | 2.31 | -2.46 | 1.64 | .70 [.67, .73] |
| Phi 9 | -4.36 | 2.08 | -2.84 | 1.55 | .67 [.63, .70] |
| Phi 10 | -3.27 | 1.76 | -2.48 | 1.35 | .60 [.57, .63] |
| LZ76 | 13.37 | 1.09 | 12.56 | 0.91 | .65 [.62, .68] |
| gzip | 27.45 | 1.24 | 27.67 | 0.79 | .53 [.50, .56] |
| Complexity 2 | 7.93 | 0.00 | 7.93 | 0.00 | .73 [.70, .76] |
| Complexity 3 | 11.89 | 0.02 | 11.87 | 0.02 | .73 [.70, .76] |
| Complexity 4 | 16.22 | 0.09 | 16.11 | 0.07 | .80 [.78, .83] |
| Complexity 5 | 20.59 | 0.16 | 20.37 | 0.13 | .83 [.81, .86] |
| Complexity 6 | 24.96 | 0.25 | 24.61 | 0.21 | .84 [.82, .86] |
| Complexity 7 | 29.37 | 0.38 | 28.85 | 0.30 | .85 [.82, .87] |
| Complexity 8 | 33.80 | 0.52 | 33.08 | 0.41 | .86 [.84, .88] |
| Complexity 9 | 38.26 | 0.68 | 37.30 | 0.54 | .86 [.84, .89] |
| Complexity 10 | 42.67 | 0.85 | 41.45 | 0.67 | .87 [.85, .89] |
| BDM 2 | 135.87 | 11.81 | 135.79 | 10.24 | .52 [.49, .55] |
| BDM 3 | 209.56 | 11.82 | 211.38 | 5.57 | .53 [.50, .56] |
| BDM 4 | 273.75 | 12.50 | 273.55 | 2.79 | .66 [.63, .69] |
| BDM 5 | 328.14 | 13.28 | 325.84 | 2.21 | .81 [.79, .84] |
| BDM 6 | 373.28 | 13.93 | 369.14 | 3.08 | .83 [.81, .85] |
| BDM 7 | 410.25 | 14.42 | 403.87 | 4.21 | .84 [.82, .86] |
| BDM 8 | 438.76 | 14.78 | 430.02 | 5.34 | .85 [.83, .88] |
| BDM 9 | 458.58 | 15.01 | 447.55 | 6.43 | .86 [.84, .88] |
| BDM 10 | 469.05 | 15.29 | 455.98 | 7.37 | .87 [.85, .89] |
| First 50 numbers of the sequences | | | | | |
| Block Entropy 2 | 4.99 | 0.22 | 5.09 | 0.12 | .61 [.58, .64] |
| Block Entropy 3 | 5.44 | 0.20 | 5.52 | 0.06 | .63 [.60, .66] |
| Block Entropy 4 | 5.50 | 0.16 | 5.55 | 0.02 | .62 [.59, .65] |
| Block Entropy 5 | 5.50 | 0.14 | 5.52 | 0.01 | .57 [.54, .60] |
| Block Entropy 6 | 5.47 | 0.12 | 5.49 | 0.00 | .53 [.50, .56] |
| Block Entropy 7 | 5.45 | 0.10 | 5.46 | 0.00 | .52 [.48, .55] |
| Block Entropy 8 | 5.42 | 0.09 | 5.43 | 0.00 | .51 [.48, .54] |
| Block Entropy 9 | 5.38 | 0.08 | 5.39 | 0.00 | .51 [.48, .54] |
| Block Entropy 10 | 5.35 | 0.08 | 5.36 | 0.00 | .51 [.48, .54] |
| RNG | 23.98 | 7.99 | 19.94 | 4.40 | .63 [.60, .66] |
| RNG2 | 21.21 | 7.18 | 19.07 | 4.38 | .56 [.53, .59] |
| Coupon | 15.44 | 5.61 | 25.34 | 8.96 | .80 [.78, .83] |
| Repetition Mean | 8.40 | 0.56 | 7.27 | 0.58 | .87 [.85, .89] |
| Repetition Median | 7.79 | 1.11 | 5.38 | 0.97 | .90 [.88, .91] |
| Repetition Mode | 6.40 | 2.66 | 2.35 | 1.66 | .85 [.82, .87] |
| Null Score | 57.25 | 4.58 | 55.09 | 3.16 | .59 [.56, .63] |
| Adjacency Asc | 10.39 | 7.32 | 9.67 | 4.19 | .51 [.48, .54] |
| Adjacency Desc | 11.27 | 5.45 | 9.79 | 4.17 | .56 [.52, .59] |
| Adjacency Combi | 21.66 | 9.21 | 19.47 | 5.37 | .55 [.52, .58] |
| Turning Points | 92.18 | 15.87 | 94.47 | 9.37 | .53 [.50, .56] |
| Runs | 0.82 | 1.11 | 0.73 | 0.34 | .49 [.46, .52] |
| Redundancy | 2.01 | 2.46 | 3.82 | 1.90 | .75 [.72, .77] |
| Phi 2 | -3.05 | 2.48 | -0.65 | 1.79 | .78 [.76, .81] |
| Phi 3 | -4.11 | 1.40 | -1.35 | 1.63 | .83 [.81, .86] |
| Phi 4 | -4.25 | 1.29 | -0.82 | 1.69 | .88 [.86, .90] |
| Phi 5 | -4.04 | 1.57 | -1.34 | 1.60 | .81 [.79, .84] |
| Phi 6 | -3.55 | 1.78 | -0.72 | 1.59 | .80 [.78, .83] |
| Phi 7 | -3.28 | 1.87 | -1.33 | 1.49 | .73 [.70, .76] |
| Phi 8 | -2.72 | 1.90 | -0.82 | 1.41 | .71 [.69, .74] |
| Phi 9 | -2.47 | 1.77 | -1.20 | 1.31 | .66 [.63, .69] |
| Phi 10 | -1.85 | 1.60 | -1.02 | 1.29 | .61 [.58, .64] |
| LZ76 | 25.37 | 1.84 | 25.29 | 1.17 | .54 [.51, .58] |
| gzip | 44.12 | 1.71 | 43.74 | 1.03 | .58 [.55, .61] |
| Complexity 2 | 7.93 | 0.00 | 7.93 | 0.00 | .80 [.77, .82] |
| Complexity 3 | 11.89 | 0.02 | 11.87 | 0.01 | .80 [.77, .82] |
| Complexity 4 | 16.22 | 0.08 | 16.11 | 0.05 | .87 [.85, .90] |
| Complexity 5 | 20.58 | 0.15 | 20.36 | 0.08 | .90 [.88, .92] |
| Complexity 6 | 24.94 | 0.23 | 24.60 | 0.13 | .90 [.88, .92] |
| Complexity 7 | 29.34 | 0.34 | 28.84 | 0.18 | .91 [.89, .93] |
| Complexity 8 | 33.76 | 0.46 | 33.06 | 0.25 | .91 [.89, .93] |
| Complexity 9 | 38.20 | 0.59 | 37.27 | 0.32 | .91 [.90, .93] |
| Complexity 10 | 42.59 | 0.74 | 41.43 | 0.39 | .92 [.90, .93] |
| BDM 2 | 282.90 | 28.32 | 296.25 | 19.38 | .59 [.57, .63] |
| BDM 3 | 533.51 | 42.42 | 552.70 | 15.29 | .63 [.60, .66] |
| BDM 4 | 745.71 | 47.31 | 754.63 | 7.08 | .62 [.59, .65] |
| BDM 5 | 936.46 | 51.10 | 936.31 | 4.39 | .62 [.59, .66] |
| BDM 6 | 1114.48 | 53.97 | 1107.03 | 5.70 | .84 [.82, .87] |
| BDM 7 | 1284.09 | 56.35 | 1268.76 | 8.07 | .89 [.87, .91] |
| BDM 8 | 1445.45 | 58.86 | 1421.64 | 10.63 | .90 [.89, .92] |
| BDM 9 | 1598.52 | 61.05 | 1565.49 | 13.33 | .91 [.89, .93] |
| BDM 10 | 1740.93 | 63.05 | 1698.50 | 16.02 | .91 [.89, .93] |
| First 100 numbers of the sequences | | | | | |
| Block Entropy 2 | 5.48 | 0.24 | 5.66 | 0.09 | .74 [.71, .77] |
| Block Entropy 3 | 6.33 | 0.28 | 6.49 | 0.05 | .76 [.74, .79] |
| Block Entropy 4 | 6.51 | 0.25 | 6.59 | 0.02 | .70 [.67, .73] |
| Block Entropy 5 | 6.54 | 0.22 | 6.58 | 0.01 | .66 [.62, .69] |
| Block Entropy 6 | 6.54 | 0.21 | 6.57 | 0.00 | .58 [.55, .61] |
| Block Entropy 7 | 6.53 | 0.19 | 6.55 | 0.00 | .54 [.51, .57] |
| Block Entropy 8 | 6.52 | 0.18 | 6.54 | 0.00 | .52 [.49, .56] |
| Block Entropy 9 | 6.50 | 0.17 | 6.52 | 0.00 | .52 [.49, .55] |
| Block Entropy 10 | 6.49 | 0.16 | 6.51 | 0.00 | .51 [.48, .54] |
| RNG | 32.21 | 6.60 | 27.15 | 2.42 | .76 [.73, .79] |
| RNG2 | 29.77 | 5.70 | 26.60 | 2.37 | .67 [.64, .71] |
| Coupon | 15.84 | 5.68 | 25.46 | 5.71 | .89 [.86, .91] |
| Repetition Mean | 8.72 | 0.28 | 8.19 | 0.28 | .87 [.85, .90] |
| Repetition Median | 7.96 | 0.89 | 5.85 | 0.73 | .90 [.88, .92] |
| Repetition Mode | 6.48 | 2.58 | 2.05 | 1.33 | .87 [.84, .89] |
| Null Score | 35.95 | 7.25 | 29.75 | 3.77 | .72 [.69, .75] |
| Adjacency Asc | 10.64 | 6.69 | 9.61 | 2.93 | .53 [.50, .56] |
| Adjacency Desc | 11.40 | 4.68 | 9.87 | 2.91 | .57 [.53, .60] |
| Adjacency Combi | 22.05 | 8.77 | 19.48 | 3.99 | .57 [.54, .60] |
| Turning Points | 92.00 | 14.76 | 94.53 | 6.39 | .54 [.51, .57] |
| Runs | 0.95 | 1.80 | 0.73 | 0.21 | .51 [.47, .54] |
| Redundancy | 1.29 | 1.38 | 1.88 | 0.91 | .68 [.65, .71] |
| Phi 2 | -2.88 | 2.59 | -0.31 | 1.25 | .84 [.81, .86] |
| Phi 3 | -3.78 | 1.51 | -0.66 | 1.20 | .90 [.87, .91] |
| Phi 4 | -4.01 | 1.15 | -0.42 | 1.23 | .93 [.92, .95] |
| Phi 5 | -3.77 | 1.35 | -0.71 | 1.20 | .88 [.86, .90] |
| Phi 6 | -3.24 | 1.53 | -0.37 | 1.18 | .87 [.85, .89] |
| Phi 7 | -2.66 | 1.58 | -0.72 | 1.14 | .77 [.74, .79] |
| Phi 8 | -2.16 | 1.63 | -0.34 | 1.09 | .74 [.72, .77] |
| Phi 9 | -1.75 | 1.56 | -0.65 | 1.00 | .68 [.65, .71] |
| Phi 10 | -1.18 | 1.31 | -0.49 | 1.02 | .63 [.60, .66] |
| LZ76 | 42.52 | 3.42 | 43.80 | 1.45 | .59 [.56, .62] |
| gzip | 65.96 | 3.07 | 66.14 | 0.93 | .43 [.40, .46] |
| Complexity 2 | 7.93 | 0.00 | 7.93 | 0.00 | .84 [.82, .87] |
| Complexity 3 | 11.89 | 0.02 | 11.87 | 0.01 | .84 [.82, .87] |
| Complexity 4 | 16.22 | 0.08 | 16.10 | 0.03 | .91 [.89, .93] |
| Complexity 5 | 20.58 | 0.15 | 20.36 | 0.06 | .92 [.90, .94] |
| Complexity 6 | 24.93 | 0.23 | 24.59 | 0.09 | .92 [.90, .94] |
| Complexity 7 | 29.32 | 0.34 | 28.83 | 0.13 | .92 [.91, .94] |
| Complexity 8 | 33.74 | 0.47 | 33.05 | 0.17 | .93 [.92, .95] |
| Complexity 9 | 38.17 | 0.60 | 37.26 | 0.22 | .94 [.92, .95] |
| Complexity 10 | 42.55 | 0.74 | 41.41 | 0.27 | .94 [.93, .95] |
| BDM 2 | 425.82 | 45.56 | 464.63 | 23.32 | .72 [.69, .75] |
| BDM 3 | 1019.44 | 105.61 | 1091.99 | 28.89 | .76 [.74, .79] |
| BDM 4 | 1510.86 | 127.10 | 1550.98 | 14.81 | .64 [.61, .67] |
| BDM 5 | 1938.47 | 143.43 | 1952.85 | 7.69 | .54 [.50, .57] |
| BDM 6 | 2339.94 | 158.56 | 2336.34 | 8.53 | .71 [.68, .74] |
| BDM 7 | 2731.34 | 173.20 | 2709.74 | 11.93 | .89 [.87, .91] |
| BDM 8 | 3113.62 | 186.98 | 3073.67 | 15.86 | .92 [.90, .93] |
| BDM 9 | 3487.51 | 199.40 | 3427.98 | 20.08 | .93 [.91, .94] |
| BDM 10 | 3848.12 | 210.31 | 3768.56 | 24.47 | .93 [.92, .95] |
| Complete sequences (200 numbers) | | | | | |
| Block Entropy 2 | 5.79 | 0.27 | 6.01 | 0.05 | .87 [.84, .89] |
| Block Entropy 3 | 7.12 | 0.38 | 7.37 | 0.05 | .89 [.88, .91] |
| Block Entropy 4 | 7.46 | 0.36 | 7.59 | 0.02 | .84 [.82, .87] |
| Block Entropy 5 | 7.54 | 0.33 | 7.61 | 0.01 | .77 [.74, .80] |
| Block Entropy 6 | 7.55 | 0.30 | 7.61 | 0.00 | .70 [.67, .73] |
| Block Entropy 7 | 7.56 | 0.28 | 7.60 | 0.00 | .59 [.56, .62] |
| Block Entropy 8 | 7.56 | 0.27 | 7.59 | 0.00 | .55 [.52, .59] |
| Block Entropy 9 | 7.55 | 0.26 | 7.58 | 0.00 | .54 [.51, .57] |
| Block Entropy 10 | 7.55 | 0.24 | 7.58 | 0.00 | .53 [.50, .56] |
| RNG | 40.76 | 5.71 | 35.87 | 1.12 | .88 [.86, .90] |
| RNG2 | 38.89 | 4.80 | 35.53 | 1.12 | .83 [.81, .86] |
| Coupon | 16.00 | 4.15 | 25.46 | 4.01 | .92 [.90, .93] |
| Repetition Mean | 8.86 | 0.22 | 8.62 | 0.13 | .86 [.84, .88] |
| Repetition Median | 8.05 | 0.79 | 6.11 | 0.53 | .94 [.92, .95] |
| Repetition Mode | 6.50 | 2.52 | 1.73 | 1.01 | .90 [.88, .92] |
| Null Score | 17.90 | 8.93 | 8.64 | 2.87 | .83 [.80, .85] |
| Adjacency Asc | 10.73 | 6.32 | 9.70 | 2.09 | .52 [.49, .55] |
| Adjacency Desc | 11.38 | 4.06 | 9.96 | 2.09 | .59 [.56, .62] |
| Adjacency Combi | 22.11 | 8.41 | 19.67 | 2.85 | .58 [.55, .61] |
| Turning Points | 91.85 | 14.10 | 94.62 | 4.48 | .54 [.51, .57] |
| Runs | 1.05 | 3.22 | 0.73 | 0.15 | .52 [.49, .55] |
| Redundancy | 0.95 | 1.13 | 0.93 | 0.46 | .44 [.41, .47] |
| Phi 2 | -2.73 | 2.73 | -0.17 | 0.89 | .88 [.86, .90] |
| Phi 3 | -3.56 | 1.59 | -0.32 | 0.86 | .92 [.90, .94] |
| Phi 4 | -3.86 | 1.11 | -0.24 | 0.89 | .96 [.94, .97] |
| Phi 5 | -3.54 | 1.26 | -0.34 | 0.87 | .94 [.92, .95] |
| Phi 6 | -3.06 | 1.33 | -0.16 | 0.84 | .91 [.90, .93] |
| Phi 7 | -2.38 | 1.34 | -0.41 | 0.82 | .83 [.81, .86] |
| Phi 8 | -1.87 | 1.43 | -0.14 | 0.82 | .79 [.77, .82] |
| Phi 9 | -1.31 | 1.45 | -0.33 | 0.81 | .70 [.67, .73] |
| Phi 10 | -0.77 | 1.11 | -0.22 | 0.78 | .63 [.59, .66] |
| LZ76 | 72.83 | 6.64 | 76.75 | 1.75 | .73 [.70, .75] |
| gzip | 108.96 | 6.93 | 111.12 | 1.08 | .64 [.61, .67] |
| Complexity 2 | 7.93 | 0.00 | 7.93 | 0.00 | .88 [.86, .90] |
| Complexity 3 | 11.89 | 0.02 | 11.87 | 0.01 | .88 [.85, .90] |
| Complexity 4 | 16.21 | 0.08 | 16.10 | 0.02 | .92 [.91, .94] |
| Complexity 5 | 20.57 | 0.15 | 20.36 | 0.04 | .93 [.92, .95] |
| Complexity 6 | 24.92 | 0.24 | 24.59 | 0.06 | .93 [.92, .95] |
| Complexity 7 | 29.31 | 0.36 | 28.82 | 0.09 | .94 [.92, .95] |
| Complexity 8 | 33.72 | 0.50 | 33.05 | 0.12 | .94 [.92, .95] |
| Complexity 9 | 38.14 | 0.65 | 37.25 | 0.15 | .94 [.93, .95] |
| Complexity 10 | 42.51 | 0.80 | 41.40 | 0.19 | .94 [.93, .96] |
| BDM 2 | 555.89 | 57.84 | 615.61 | 18.06 | .83 [.80, .85] |
| BDM 3 | 1843.90 | 228.74 | 2068.56 | 51.68 | .89 [.87, .91] |
| BDM 4 | 2978.33 | 309.34 | 3127.96 | 28.85 | .79 [.77, .82] |
| BDM 5 | 3914.87 | 356.00 | 3984.43 | 13.37 | .60 [.57, .64] |
| BDM 6 | 4773.71 | 396.97 | 4795.12 | 12.46 | .46 [.43, .50] |
| BDM 7 | 5611.89 | 437.44 | 5591.98 | 17.06 | .80 [.77, .82] |
| BDM 8 | 6437.00 | 476.95 | 6377.92 | 22.82 | .92 [.91, .94] |
| BDM 9 | 7253.01 | 513.77 | 7152.85 | 29.04 | .93 [.91, .94] |
| BDM 10 | 8050.52 | 547.28 | 7908.17 | 35.52 | .93 [.91, .94] |

*Note.* Asc = Ascending. Desc = Descending. Combi = Combined. Numbers at the end of a measure indicate the block size used in the calculation. Values in square brackets indicate empirical confidence limits (95%).
